# 349. The impact of Vancomycin dosing by area under the curve over minimal inhibitory concentration (AUC/MIC) versus Trough levels on cumulative Vancomycin dosage and incidence of Acute Kidney Injury (AKI) in patients admitted to intensive care units (ICU)

**DOI:** 10.1093/ofid/ofad500.420

**Published:** 2023-11-27

**Authors:** Aniruddha Sahu, Kevin K Wang, Philip Jurasinski, Salil Kulkarni, Sayo Weihs, Masako Mizusawa, David Bamberger

**Affiliations:** University of Missouri Kansas City, kansas city, Missouri; University of Missouri, Kansas City, Kansas city, Missouri; University of Missouri, Kansas City, Kansas city, Missouri; University of Missouri, Kansas City, Kansas city, Missouri; University Health, Kansas city, Missouri; University of Missouri-Kansas City, Kansas City, Missouri; University of Missouri-Kansas city, Kansas City, Missouri

## Abstract

**Background:**

Higher Vancomycin trough levels (15-20 mcg/ml) are associated with increased risk of AKI. IDSA recommends dosing Vancomycin as per AUC/MIC reduces the risk of nephrotoxicity. The aim of this study was to determine if AUC/MIC dosing could reduce the incidence of nephrotoxicity patients in admitted to intensive care unit (ICU). This has not been previously studied.

**Methods:**

This was a retrospective pre and post chart review study including all admitted patients to ICU at Truman Medical Center Hospital Hill, Kansas City, Missouri from 10/1/2020 to 10/1/2021. The pre intervention period (trough monitoring) was 10/1/2020-3/29/2021.The post intervention period was 4/1/2021-10/1/2021, included patients who received Vancomycin dosing via using a Bayesian derived AUC monitoring software program targeting AUC of 400-600 mg*hour/L (Insight Rx). All patients who were admitted to ICU and received Vancomycin for >48 hours were included. Exclusion criteria includes Vancomycin use for < 48 hours, end stage renal disease and who received continuous renal replacement therapy within 48 hours of starting Vancomycin.

**Results:**

119 patients received Vancomycin dosing by AUC/MIC and 122 patients by Vancomycin trough. Patients received 23.51 ± 2.13 mg/kg/day(mean ± standard error) Vancomycin in AUC/MIC and 25.28 ± 1.71 mg/kg/day in trough group (p = 0.18). The incidence of AKI was 25.21%(n=30) in AUC/MIC group and 17.21%(n=21) in trough group (p = 0.13). Vancomycin was used for 5.61 ± 0.68 days in AUC/MIC group and 4.92 ± 0.48 days in trough group (p = 0.10). The incidence of AKI in patients who received concomitant intravenous contrast was 26.22%(n=16) in AUC/MIC group and 10.90%(n=6) in trough group (p = 0.04*). Average length of stay (LOS) in ICU was 11.56 ± 2.65 days in AUC/MIC group and 8.66 ± 1.61 days in trough group (p = 0.06). All-cause mortality during hospitalization was 29.41%(n=35) in AUC/MIC group and 22.13%(n=27) in trough group (p = 0.20). AKI was noted in 19.82% patients receiving Piperacillin tazobactam and Vancomycin versus 22.4% receiving Vancomycin without Piperacillin tazobactam(p = 0.63).

**Figure:**
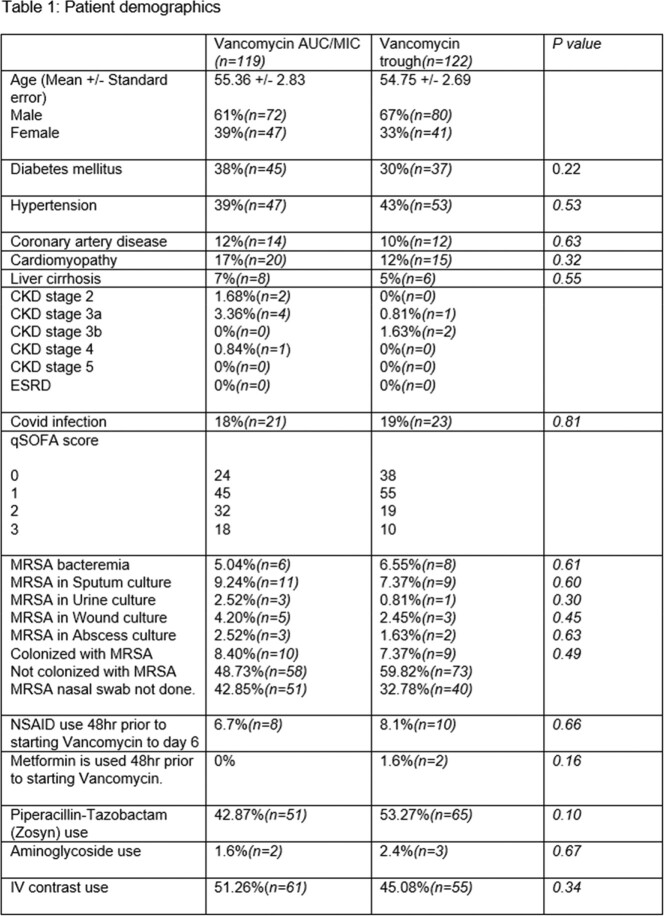

**Figure:**
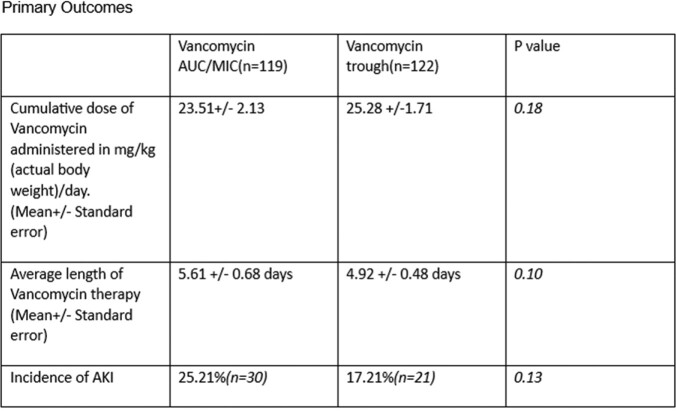

**Figure:**
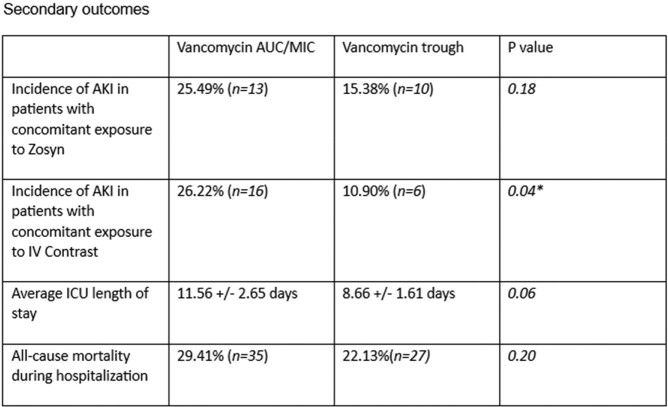

**Conclusion:**

There was no difference in cumulative vancomycin dose, incidence of AKI, LOS and All-cause mortality during the hospitalization while using Vancomycin dosing by AUC/MIC vs trough in ICU patients.

**Disclosures:**

**All Authors**: No reported disclosures

